# Enhanced Thermoelectric Conversion Efficiency of CVD Graphene with Reduced Grain Sizes

**DOI:** 10.3390/nano8070557

**Published:** 2018-07-22

**Authors:** Gyumin Lim, Kenneth David Kihm, Hong Goo Kim, Woorim Lee, Woomin Lee, Kyung Rok Pyun, Sosan Cheon, Phillip Lee, Jin Young Min, Seung Hwan Ko

**Affiliations:** 1 School of Mechanical and Aerospace Engineering, Seoul National University, Seoul 08826, Korea; gyumin37@snu.ac.kr (G.L.); honggu99@snu.ac.kr (H.G.K.); lwr0516@snu.ac.kr (Woorim L.); woomin@snu.ac.kr (Woomin L.); kyungrok.pyun@snu.ac.kr (K.R.P.); cjsthtks@gmail.com (S.C.); maxko@snu.ac.kr (S.H.K.); 2 Department of Mechanical, Aerospace, and Biomedical Engineering, University of Tennessee, Knoxville, TN 37996, USA; 3 Korea Institute of Science and Technology, Seoul 02792, Korea; phillip@kist.re.kr; 4 School of Mechanical Engineering, Korea University, Seoul 02841, Korea; guid12@korea.ac.kr; 5 Institute of Advanced Machinery and Design (SNU-IAMD), Seoul National University, Gwanak-ro, Gwanak-gu, Seoul 08826, Korea

**Keywords:** thermoelectric conversion efficiency, CVD graphene, grain sizes, FET 4-point measurements, electrical conductivity, Seebeck coefficient

## Abstract

The grain size of CVD (Chemical Vapor Deposition) graphene was controlled by changing the precursor gas flow rates, operation temperature, and chamber pressure. Graphene of average grain sizes of 4.1 µm, 2.2 µm, and 0.5 µm was synthesized in high quality and full coverage. The possibility to tailor the thermoelectric conversion characteristics of graphene has been exhibited by examining the grain size effect on the three elementary thermal and electrical properties of *σ*, *S*, and *k*. Electrical conductivity (*σ*) and Seebeck coefficients (*S*) were measured in a vacuum for supported graphene on SiO_2_/Si FET (Field Effect Transistor) substrates so that the charge carrier density could be changed by applying a gate voltage ($V_{G}$). Mobility (*µ*) values of 529, 459, and 314 cm^2^/V·s for holes and 1042, 745, and 490 cm^2^/V·s for electrons for the three grain sizes of 4.1 µm, 2.2 µm, and 0.5 µm, respectively, were obtained from the slopes of the measured *σ* vs. $V_{G}$ graphs. The power factor (PF), the electrical portion of the thermoelectric figure of merit (ZT), decreased by about one half as the grain size was decreased, while the thermal conductivity (*k*) decreased by one quarter for the same grain decrease. Finally, the resulting ZT increased more than two times when the grain size was reduced from 4.1 µm to 0.5 µm.

## 1. Introduction

The thermoelectric effect enables direct energy conversions between temperature and electric voltage differences. When a temperature gradient is applied, the momentum difference between charge carriers causes them to shift to one side, yielding voltage potential inside the materials. Since it allows for the conversion of wasted heat into electrical energy, having control over the thermoelectric effect would give rise to one of the most promising sources of renewable energy, because the eco-friendly generation of electrical energy only requires a temperature difference to reuse the wasted heat energy.

Graphene has high potential for becoming a thermoelectric material due to its high electrical conductivity and Seebeck coefficient. Its high thermal conductivity, however, has prevented graphene from being used as a thermoelectric material in reality. In order to enhance graphene’s thermoelectric properties, many ideas have been proposed aimed at lowering its thermal conductivity, including defect controlling [1] and management of grain size [2,3]. Among the various proposals, controlling grain size seems to be a highly viable way to handle the carriers’ scattering of graphene, because it does not add artificial disorder, but only modifies the preexisting grain boundaries of CVD graphene. To the authors’ knowledge, thus far no study has attempted to characterize the figure of merit ($\mathrm{ZT}=\frac{\sigma S^{2}T}{k}$) for graphene with controlled grain sizes. Measurements have been made for electrical conductivity with controlled grain sizes [3]; however, no measurements on Seebeck coefficients have been reported. In our previous study, we have experimentally verified a decrement of thermal conductivity by controlling the polycrystalline graphene domain sizes [2], which can lead to the possibility of ZT enhancement of CVD graphene.

In this paper, as there is still a need for examining the ZT values, we focus on measuring both the electrical conductivity (*σ*) and the Seebeck coefficients (*S*). Graphene was synthesized on Cu foil using the LPCVD (Low Pressure Chemical Vapor Deposition) method, which allows for the control of the grain sizes, and then subsequently transferred onto a SiO_2_/Si (450 nm/525 µm) FET substrate. The electrical properties (*σ* and *S*) of graphene were measured using the four-point technique for three different grain sizes of 0.5 µm, 2.2 µm, and 4.1 µm, while the charge carrier density was controlled by varying the gate voltage levels.

## 2. Materials and Methods

**Figure 1:**
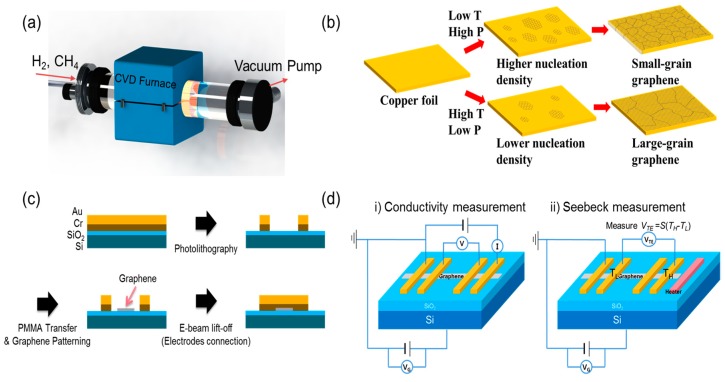
(**a**) Schematic of CVD graphene synthesis system, (**b**) grain size control by synthesis temperature and pressure variations, (**c**) fabrication process diagram for the Field Effect Transistor (FET) substrate with electrode/graphene sample laid down, and (**d**) schematic of the 4-point measurement layout.

While the details on the CVD graphene sample synthesis have been discussed in our group’s previous publication [2], we hereby present the essential description of the technique to provide comprehensive idea of how the different grain sizes were devised in CVD synthesis. Graphene was synthesized from the CVD system by ScienTech Inc. (Figure 1a), in which CH_4_ was used as the carbon source and H_2_ was used as cocatalyst to dissociate H atoms upon their detachment from CH_4,_ as well as to etch the weak carbon-carbon bonds and eliminate multiple layers [4]. Thus, hydrogen acts to etch the weak C—C bond and tends to eliminate multiple layers. The 25 µm-thick Cu foil with 99.999% purity (Alfa Aesar Inc.) was used as both a catalyst and substrate. The grain size of graphene was controlled by comprehensively changing the CH_4_ to H_2_ ratio, temperature (*T*), and pressure (*P*) of the chamber. Details of the synthesis conditions are shown in Table 1.

CVD graphene with smaller grain sizes was synthesized at low *T* and under high *P* to make the nucleation density higher, whereas graphene with larger grain sizes was synthesized at high *T* and low *P* to make the nucleation density lower, as depicted in Figure 1b. For both the 4.1 µm and 2.2 µm graphene samples, graphene was synthesized following a two-step process: first, flow rates for CH_4_ and H_2_ were set relatively low so that graphene could grow slowly with enlarged grain sizes; second, the gas flow rates were set high in order to supply enough of a carbon source to ensure the full coverage of graphene. For the 0.5 µm graphene, it was synthesized in a single step with high gas flow rates so that the graphene would grow rapidly while the grain size stayed small. This also enabled full coverage of graphene.

The FET substrate was fabricated through lithography, as schematically shown in Figure 1c. It was designed to measure electrical conductivity and Seebeck coefficients in the same substrate by using a micro-heater and electrodes to detect electrical potentials. The micro-heater/electrodes were made of 200 nm thick Au laid on a 20 nm thick Cr contact layer, which were patterned through photolithography on a Si substrate with a thermally oxidized, 450 nm-thick SiO_2_ layer on the top surface. Graphene was patterned between the electrodes via photolithography, and the electrodes were connected above the graphene through e-beam lithography.

The 4-point measurement (Figure 1d) is an electrical measuring technique that uses separate pairs of current-carrying and voltage-sensing electrodes to make more accurate measurements, as compared to the conventional two-point sensing. The key advantage of the 4-point method is that the separation of current- and voltage-electrodes eliminates the errors caused by the wiring and contact resistances.

After fabricating the FET substrates, graphene was transferred onto it using the well-established PMMA method. CVD graphene is naturally *p*-doped upon exposure to the oxygen and hydrogen atoms in air. Since we wanted to observe the Dirac point, we needed to minimize the *p*-doping effect by means of vacuum annealing. Annealing under excessively high temperature and/or overly long duration is known to attach graphene to the substrate too strongly, so much so that it degrades the quality of graphene [5,6,7]. Accordingly, we opted to anneal graphene only for 2 h at 250 °C so that the *p*-doping effect would be sufficiently reduced without degrading the graphene sample.

**Figure 2:**
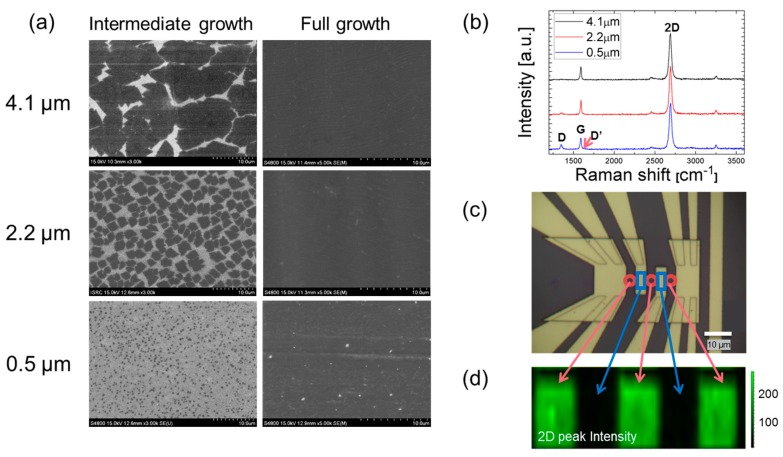
(**a**) SEM images of graphene growth on Cu foil for three different grain sizes. (**b**) Raman spectra of graphene samples laid on the FET substrate. (**c**) Optical image of the 4-point electrodes with graphene sample integrated. (**d**) 2D peak Raman mapping of graphene to distinguish the exposed graphene regions (green) from the electrode-covered regions (black).

The SEM image was taken once during the intermediate growth of grains and again after the full growth on the Cu foil (Figure 2a). The less dense seeding of graphene provides bigger graphene islands that eventually grow into larger grains. Mild dry annealing was used to oxidize Cu foils along the grain boundaries to identify grain sizes of fully grown graphene [2]. Then, the images were digitized to enhance the contrast and processed to measure the grain sizes for each of the three samples: 4.095 ± 0.468 µm, 2.224 ± 0.258 µm, and 0.524 ± 0.059 µm, in which the rms error with 95% confidence intervals accounts for up to 100 data points, In which the total graphene area was divided by the total number of grains to determine the average grain sizes of the three tested samples: 4.1 µm, 2.2 µm, and 0.5 µm. The 2D and G peaks in the Raman spectra (Figure 2b) are located near 2700 and 1600 cm^−1^, respectively, and the 2D/G peak ratio is greater than two for all three samples, indicating that high quality single layer graphene was properly transferred onto the SiO_2_/Si substrate [8,9,10,11,12]. Also, the measured absorption of less than 3% for the graphene suspended on the hole patterned substrate confirms the consistency with the absorption range of mono-layered CVD graphene [2]. For the sample with the smallest grain size, the D peak starts to appear due to the enhanced defects or atomic irregularities associated with the increased grain boundaries. The D/D’ peak intensity ratio of about 3.5 also implies that the D peak appearance can be attributed to the boundary defects [13]. In Figure 2c, the microscopic image indicates the well-fabricated FET 4-point electrodes. The 2D peak Raman mapping (Figure 2d) exhibits the uniform 2D peak intensity distribution in the exposed graphene area marked in green (corresponding to the red circles in Figure 2c), whereas the alternative areas marked in black (corresponding to the blue squares) do not show any intense 2D peaks due to the Au electrodes’ coverage over the graphene. The uniform 2D peak intensity at the exposed graphene areas between the electrodes confirms the continuous and uniform-quality of the tested graphene.

## 3. Results and Discussion

**Figure 3:**
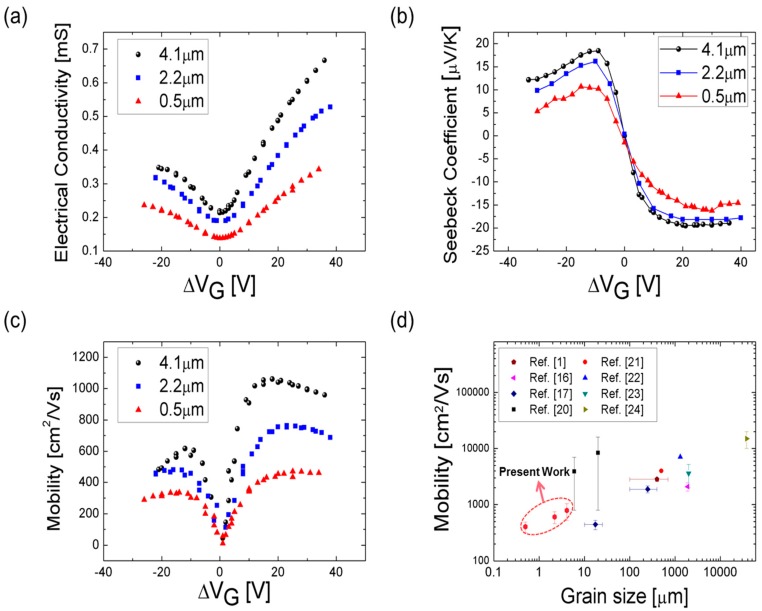
(**a**) Electrical conductivity for the three grain sizes of 4.1 µm, 2.2 µm, and 0.5 µm as functions of the gate voltage sweep. (**b**) Seebeck coefficient for the three grain sizes as functions of the gate voltage sweep. (**c**) Mobility for the three grain sizes as functions of the gate voltage. (**d**) Measured mobility data compared with published data for varied grain sizes.

The measured electrical conductivities (σ) and Seebeck coefficients (*S*) are shown in Figure 3a,b, respectively. We determined Dirac points with minimum conductivity values [14,15,16,17], and both graphs are plotted as functions of $\Delta V_{G}$ (≡$V_{G}-V_{G,\;Dirac}$, in which $V_{G}$ is the gate voltage applied and $V_{G,\;Dirac}$ is the gate voltage at the Dirac point). So, the minimum conductivity values are perfectly centered in $\Delta V_{G}$ = 0 V. Asymmetry transfer characteristic between electron and hole conduction is due to the Cr contact layer, which acts as an n-dopant metal with lower work function than the graphene that lower hole conductivity [18]. The corresponding absolute maximum values of the Seebeck coefficient were about 19.5 µV/K, 18.2 µV/K, and 16.2 µV/K (Figure 3b), respectively, in a positive $\Delta V_{G}$ range. The electrical mobility (*µ*) of graphene (Figure 3c) was then determined from the slope of the electrical conductivity, which is given by

(1) $$\sigma \left(V_{G}\right)=C_{SiO_{2}}\mu \left|V_{G}-V_{G,Dirac}\right|+\sigma_{Dirac},$$

in which *σ* is the electrical conductivity, $C_{SiO_{2}}$ is the oxide capacitance per unit area, and *µ* is the electrical mobility [19]. The oxide capacitance per unit area can be calculated as $C_{SiO_{2}}=\varepsilon_{SiO_{2}}\times \frac{1}{d}=3.9\times \left(8.85\times 10^{-12}\right)\times \frac{1}{450\times 10^{-9}}=7.67\times 10^{-5}F/m^{2}$, in which $\varepsilon_{SiO_{2}}$ is the permittivity of the oxide and d is the oxide thickness. Effective mobility values of graphene were measured to be 529 cm^2^/V·s, 459 cm^2^/V·s, and 314 cm^2^/V·s for holes and 1042 cm^2^/V·s, 745 cm^2^/V·s, and 490 cm^2^/V·s for electrons for the grain sizes of 4.1 µm, 2.2 µm, and 0.5 µm, respectively. Measured mobility data for relatively larger grains by other research groups [1,16,17,20,21,22,23,24] are also shown in Figure 3d. A gradual increase of the electrical mobility is shown with increasing grain sizes, at the ratio of one order-of-magnitude increase of mobility to nearly four orders-of-magnitude increase of the grain size.

Previously published Seebeck coefficient data are summarized in Table 2 [1,15,25,26,27,28,29,30,31]. Only one group [1] specified actual grain size of their tested sample, but only to show the uncontrolled range from 100 to 700 µm. Indeed, this work examined the plasma-created defects, which inherently creates a wide range of defect shapes and sizes. While the grain size effect on the Seebeck coefficients was not examined in these studies, the reported data ranges from 10 to 100 µV/K, depending on different graphene sample preparations and post-treatments. It is known that the residual carrier density induced by charged impurities has significant effects on Seebeck coefficients near the Dirac point, thus consequently affecting the maximum values of Seebeck coefficients [32]. This also implies that inherent charged impurities induced during the fabrication process can result in variations of measured Seebeck coefficients. Our measured range of Seebeck coefficients of 16–20 µV/K is smaller than the 55 µV/K measured for the case of the 300 µm grain size [1], which uniquely specifies the grain size of their graphene samples.

**Figure 4:**
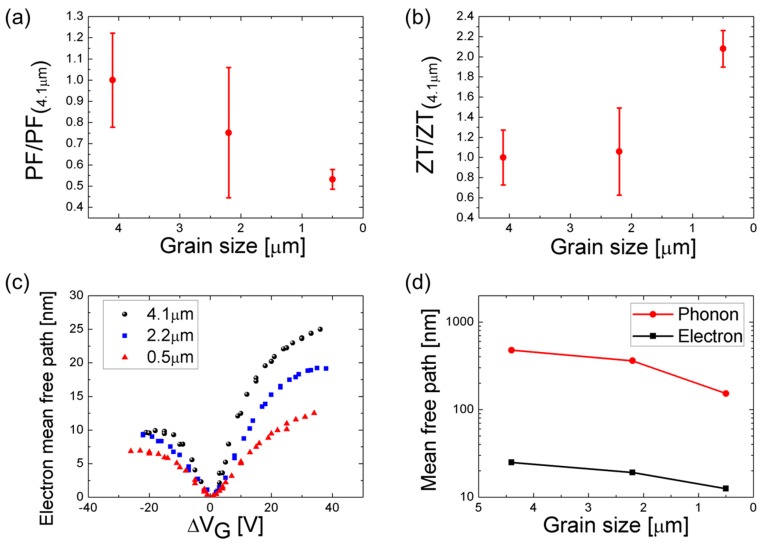
(**a**) Power factor dependence on the grain sizes of graphene. (**b**) Gradually increasing ZT with decreasing grain sizes. (**c**) Electron mean free path as a function of gate voltage for the graphene of grain sizes 4.1 µm, 2.2 µm, and 0.5 µm. (**d**) Phonon and electron mean-free-path of graphene of grain sizes 4.1 µm, 2.2 µm, and 0.5 µm.

The thermoelectric figure-of-merit ($\mathrm{ZT}=\frac{\sigma S^{2}T}{k}$) is determined from the presently measured σ, *S*, and *T*, together with the thermal conductivity *k* data measured for a similar configuration by our group’s previous report, as 2660, 1890, and 680 W/m·K for grain sizes 4.1 µm, 2.2 µm, and 0.5 µm, respectively [2]. The normalized power factor (PF = $\sigma S^{2}T$, Figure 4a) shows a slower decrease with decreasing grain size than the corresponding *k* decrease: the PF decreases to 1/2, while *k* decreases to 1/4. Consequently, when the grain size is reduced from 4.1 µm to 0.5 µm, the ZT value (Figure 4b) increases by approximately two times. The corresponding ZT values are 0.55 × 10^−4^, 0.58 × 10^−4^, and 1.13 × 10^−4^ at room temperature (*T* = 300 K). The detrimental effect of the grain size on *k* becomes more significant when the grain size is comparable to the phonon mean-free-path (MFP) of about 800 nm [2]. Also, σ shows a predominantly decreasing pattern for grain sizes smaller than 800 nm [3]. In other words, the grain size effect on ZT is less pronounced when the grain sizes are 4.1 µm or 2.2 µm, but shows a dramatic increase for the sub-micron grain size of 0.5 µm. The rms error bars with 95% confidence intervals account for the 5–10 measurement samples for each grain size.

It is well known that the electron scattering increment at the grain boundaries reduces the electrical transport and properties [14,17,20]. The electron MFP (Figure 4c) is given by [33,34], $l_{mfp}=\left(\frac{h}{2e}\right)\mu \sqrt{\frac{n}{\pi }}$, in which *h* is Planck’s constant, *e* is the elementary charge, *µ* is the electrical mobility, and *n* is the charge carrier density. Mobility values obtained as functions of gate voltage in Figure 3c were used to get the electron MFP (Mean Free Path) in Figure 4c. The estimated electron MFPs converge to 24.9 nm, 19.1 nm, and 12.5 nm for the grain sizes of 4.1 µm, 2.2 µm, and the 0.5, respectively (Figure 4d). The decrements in the 2.2 µm and the 0.5 µm samples were 23.3% and 49.8%, respectively, relative to the 4.1 µm sample. The electron MFPs were reported to be in the range from 10 to 100 nm for the charge carrier density range of 10^12^–10^13^ cm^−2^, which corresponds to the gate voltage range larger than 13 V [35].

In contrast, the phonon MFP is given by a Landauer-like approach [36,37], $k\left(l_{G}\right)=G_{ball}{\left[\frac{1}{l_{G}}+\frac{2}{\pi \lambda }\right]}^{-1}$, in which *G_ball_* is the ballistic thermal conductance (~4.2 × 10^9^ W/m^2^K at room temperature), *l_G_* is the grain size, and *λ* is the phonon MFP. The estimated phonon MFP’s are 476.9 nm, 360.1 nm, and 152.4 nm for the grain sizes of 4.1 µm, 2.2 µm, and 0.5 µm, respectively (Figure 4d). The decrements in the 2.1 µm and 0.5 µm samples were 24.5% and 68.0%, respectively, from the 4.1 µm sample. The larger decrements of the phonon MFPs than the electron MFPs support the idea that decreasing grain size is more effective for enhancing the scattering rate of phonons than electrons. This is possibly the reason why graphene of reduced grain sizes shows lower decrement for electrical properties than for thermal conductivity, and thus results in an increased ZT.

## 4. Conclusions

We investigated the dependence of the thermoelectric figure of merit, ZT, on the grain size of CVD graphene. Electrical conductivity (*σ*) and Seebeck coefficients (*S*) were measured for three different grain sizes: 4.1, 2.2, and 0.5 μm, using a FET 4-point measurement technique. Since the decrement of the corresponding thermal conductivity (*k*) was larger than the decrement of the PF, more than two times the original ZT value was observed as the grain size was decreased from 4.1 μm to 0.5 μm. We have shown the possibility that ZT can be tailored by altering the grain size of graphene, which is a crucial factor in CVD graphene synthesis. Furthermore, this enhancement of the thermoelectric properties opens the possibility of graphene to be considered as a more realistic thermoelectric material.

## Figures and Tables

**Table 1:** CVD graphene synthesis conditions for three different grain sizes.

Grain Size [µm]	4.1		2.2		0.5
**Temperature [°C]**	1000		900		800
**Pressure [Torr]**	Step 1	Step 2	Step 1	Step 2	1.09
	0.19	0.30	0.37	1.08	
**Gas flow rate ratio CH_4_:H_2_ [sccm:sccm]**	30:5	60:5	80:5	200:100	200:100
**Gas flow duration [min]**	10	5	20	10	25

**Table 2:** Published list of Seebeck coefficients of CVD graphene.

Reference	Published Year	Grain Size	Seebeck Coefficient	Descriptions
Ref. [26]	2010	-	~9 µV/K @ 300 K	Linear dependence of S on T for 50 < T < 300 K
Ref. [27]	2011	-	~50 µV/K @ 500K ~30 µV/K @ 300K	Sensitivity of S to the surface charge doping by exposure to the air, N_2_O, and NH_3_
Ref. [28]	2013	-	~10 µV/K @ 300K	Linear dependence of S and electrical conductivity on T for 75 < T < 300 K
Ref. [15]	2014	-	~20 µV/K @ 150K	Observation on the large fluctuation of S near the Dirac point associated with the disorder in graphene at high magnetic field & low temperature
Ref. [29]	2015	-	~100 µV/K @ 300K	N-type doping of CVD graphene by H_2_ exposure verified by S measurement
Ref. [1]	2017	Average 300 µm (100–700)	~55 µV/K @ RT	ZT enhancement using O_2_ plasma irradiation. (ZT/ZT_0_~3)
Ref. [31]	2018	-	~30 µV/K @ RT	Estimation of electrical conductivity and Seebeck of graphene sheet and graphene nanoribbon by experimental and theoretical approach

Note: The grain size dependence of Seebeck coefficients is unavailable from any of these studies.
